# Exposome on skeletal muscle system: a mini-review

**DOI:** 10.1007/s00421-024-05515-1

**Published:** 2024-05-28

**Authors:** Cristina Purcaro, Lorenzo Marramiero, Carmen Santangelo, Danilo Bondi, Ester Sara Di Filippo

**Affiliations:** 1 Department of Neuroscience, Imaging and Clinical Sciences, University “G. d’Annunzio” Chieti-Pescara, Via dei Vestini, 31, Chieti, Italy; 2 IIM-Interuniversity Institute of Myology, Chieti, Italy

**Keywords:** Stress, Hormesis, Pollutants, Stem cells, Lifestyle

## Abstract

Exposomics is an ever-expanding field which captures the cumulative exposures to chemical, biological, physical, lifestyle, and social factors associated with biological responses. Since skeletal muscle is currently considered as the largest secretory organ and shows substantial plasticity over the life course, this reviews addresses the topic of exposome and skeletal muscle by reviewing the state-of-the-art evidence and the most intriguing perspectives. Muscle stem cells react to stressors via phosphorylated eukaryotic initiation factor 2α and tuberous sclerosis 1, and are sensible to hormetic factors via sirtuin 1. Microplastics can delay muscle regeneration via p38 mitogen-activated protein kinases and induce transdifferentiation to adipocytes via nuclear factor kappa B. Acrolein can inhibit myogenic differentiation and disrupt redox system. Heavy metals have been associated with reduced muscle strength in children. The deep study of pollutants and biological features can shed new light on neuromuscular pathophysiology. The analysis of a time-varying and dynamic exposome risk score from a panel of exposure and phenotypes of interest is promising. The systematization of hormetic factors and the role of the microbiota in modulating the effects of exposure on skeletal muscle responses are also promising. The comprehensive exposure assessment and its interactions with endogenous processes and the resulting biological effects deserve more efforts in the field of muscle health across the lifespan.

## Exposomics

The concept of exposome has been proposed to match that of genome and developing precise methods for an individual's environmental exposure. It has been defined as the highly variable and dynamic entity that encompasses life-course environmental exposures and evolves throughout the lifetime of the individual (Wild 2005). Since 2005, exposome has been extensively characterized as a new field, with scientific networks (e.g., https://humanexposomeproject.com, https://exposome.nl) and a specific journal recently launched (Miller 2021). In the nature vs nurture conundrum, what we were missing was the systematization of the latter. For capturing the essence of nurture, the definition of exposome has been extended to the following one: “the cumulative measure of environmental influences and associated biological responses throughout the lifespan, including exposures from the environment, diet, behavior, and endogenous processes” (Miller and Jones 2014). The definition of exposome, thus, may regard the exposure to several types of factors, among which physical factors, psychosocial stressors, dietary element, and toxicants penetrating through inhalation or ingestion as well as chemicals produced by physiological process, such as infections, disruption of redox system, and inflammation (Vermeulen et al. 2020).

Comprehensively, exposomics can be referred to chemical, biological, physical, lifestyle, and social factors. Specifically, agents such as toxicants, pollutants, social threats, microbial invasions, viruses, temperature, oxygen, nutrients, physical activity, psychological loads, all contribute to the biological footprint. Intersecting dynamic exposures with dynamic biological processes represents a promising challenge for providing a realistic assessment of major drivers of healthy vs unhealthy processes, providing a means to prioritize research agendas and interventions (Kalia et al. 2022).

## Exposome and human health

The exposome has a crucial role in defining human health, and becomes even more crucial when considering that early exposure and environmentally induced transgenerational alterations greatly affect behavior and disease susceptibility across lifespan through epigenetic modifications (Jirtle and Skinner 2007). The result of the complex interactions of genes, epigenetics, microorganisms, and exposome can comprehensively define the human phenome (Tian et al. 2022). Within this framework, skeletal muscle plays a key role as the largest secretory organ, with a growing body of evidence on the muscle organ cross-talk and the ever-expanding list of myokines (Mancinelli et al. 2021; Magliulo et al. 2022; Pedersen 2023). Moreover, skeletal muscle system exhibits great plasticity to exposomics factors such as physical exercise (Bathgate et al. 2018). However, integrating exposomics with skeletal muscle health and plasticity across the lifespan is a topic with a dearth of information. Moving from these perspectives, this timely review aims to summarize the recent advancements on the role of exposome on skeletal muscle system and provide future perspectives within this topic.

## Exposome to muscle: the beneficial side

The decline of the adaptive interface between functional genome and life-course environmental factors, and the exposure memory which decreases the plasticity of genome–exposome interaction modify stress-responsive cellular pathways at a cellular level (Bevere et al. 2022). Factors such as environmental stressors can affect cells among which muscle stem cells (MSCs). When stress is severe and continuous, cells activate pathways that lead to death. Otherwise, when stress stimuli do not exceed a specific threshold, cells initiate protective responses. In the latter case, MSCs exhibit two types of reactions. A first group of MSCs is involved in a regenerative response to stress stimuli, leading to the increment of the stem cell pools. The initial step of this process is general protein synthesis inhibition, essential to reduce energy requirement and to guarantee self-renewal and quiescence. At the same time, the translation of specific mRNAs involved in stress response occurs. On the other hand, the second group of MSCs with lower ability to fight stress can avoid it through spontaneous myogenic differentiation and this process is caused by numerous factors, in particular gene inactivation and exposure to environmental pollutants. Regarding gene inactivation, inhibition of phosphorylated eukaryotic initiation factor 2α (P-eIF2α) and tuberous sclerosis 1 (Tsc1) seems to lead to MSCs differentiation under stress conditions. P-eIF2α takes part in the integrated stress response, an intracellular signaling pathway that causes, among others, mRNA translation repression and RNA granule formation for stopped mRNA sequestration. Looking at Tsc1, it forms a complex with tuberous sclerosis 2 (Tsc1/Tsc2) which is activated by stress stimuli with the aim of inhibiting complex 1 of mammalian target of rapamycin (mTORC1) signaling. The inhibition of mTORC1 produces decrease of cell growth and proliferation. Hence, the inhibition of P-eIF2a and/or Tsc1 is responsible for myogenic differentiation in the second MSCs group. In light of this, only MSCs capable of reacting against stress stimuli continue to belong to the stem cell pools. Moreover, these cells under the stress stimulus leave the quiescence state and proliferate repopulating the pools involved in muscle regeneration as long as the stimulus lasts. MSCs not properly able to react against stress exit from the mentioned pools undergoing differentiation, or reaching death when the stress is too severe (Gugliuzza and Crist 2022).

A broad spectrum of chemical classes (including dietary supplements, pharmaceuticals, pollutants) and physical stressors induce hormetic responses in muscle stem cells. Some dietary supplements can diffuse from the systemic circulation to the MSCs, thus altering the niche and possibly stimulating MSCs themselves. With this regard, agents such as ferulic acid, nicotinamide and resveratrol enhance MSCs activation, proliferation and/or differentiation by upregulating Sirtuin1 (Calabrese and Calabrese 2022). The plant-derived natural products represent a key factor to positively modulate muscle inflammaging—i.e., the low-grade, chronic, systemic inflammation that occurs with aging, not ascribable to overt infections (Franceschi and Campisi 2014)—observed during sarcopenia, reducing muscle wasting and promoting muscle regeneration and protein synthesis, and improving fatigue resistance when combined with exercise training. In addition to the aforementioned, catechin, ginko biloba and soy protein act modulating key mitochondrial pathway, including the phosphatidylinositol 3-kinase (PI3K)/Akt. This results in a reduction in oxidative stress, particularly when Nrf2 upregulation occurs as a consequence of curcumin ingestion. The skeletal muscle improvement may also be reached by providing a proper intake of protein, long-chain polyunsaturated fatty acids, and vitamin D (Bagherniya et al. 2022).

## Exposome to muscle: the detrimental side

Several studies demonstrate how pollutants, which belong to the exposome, can affect human health, also exerting influence on skeletal muscles. For instance, Wang Shengchen and colleagues evaluated the effect of polystyrene microplastics on the regeneration of skeletal muscle. For this reason, they exposed male mice to two kinds of polystyrene microplastics with different diameters (1–10 µm and 50–100 µm) for 30 days observing skeletal muscle regeneration after injury with BaCl_2_. Their experiments demonstrate how microplastics exposure delays muscle regeneration, reducing muscle fiber diameter and their average cross-sectional area. In fact, microplastics are able to reduce p38 mitogen-activated protein kinases (p38 MAPK) phosphorylation inhibiting myogenic differentiation, while they increase nuclear factor kappa B (NF-κB) expression promoting adipogenic differentiation and lipid deposition. Moreover, that group showed that all the cited processes are driven by increased reactive oxygen species (ROS) production due to altered superoxide dismutase and catalase activities and reduced total antioxidant capacity. In particular, higher ROS levels appear to be coupled with NF-κB signaling via a platelet endothelial cell adhesion molecule (PECAM-1)-dependent process that is ending with the transdifferentiation of cells associated with skeletal myofibers, promoting the adipocyte phenotype. Consequently, higher oxidants levels are recorded after treatment with microplastics leading to muscle regeneration impairment (Shengchen et al. 2021). However, whether or not micro-and-nano plastics accumulate in human muscle tissue thus resulting in putative detrimental effects has yet to be demonstrated (Ali et al. 2024).

Delayed muscle regeneration can also be caused by some substances, among others acrolein that is a ubiquitous environmental toxicant. Specifically, human exposure occurs through fossil fuel combustion, inhalation of vehicle emission, dietary intake (natural water and food content, food processing such as frying and baking, pesticides), and by fermentation of the gut microbiota as endogenous source (Jiang et al. 2022). Acrolein can, indeed, significantly inhibit myogenic differentiation as shown by Huang-Jen Chen and colleagues. These authors evaluated acrolein effects through in vitro studies on C2C12 cells and in vivo studies on five-week-old male ICR mice. In vitro results highlight myogenic differentiation repression after acrolein treatment that seems to be driven by protein kinase B (Akt) phosphorylation inhibition and consequently Akt signaling inhibition in a dose-dependent manner. Akt is part of the phosphatidylinositol 3-kinase (PI3K)/Akt pathway, known to play a fundamental role in myogenesis. In addition, results obtained in vivo exhibit how acrolein causes muscle atrophy due to increased ROS levels. In fact, acrolein is a lipid peroxidation product and it is able to link glutathione, leading to reduction of cellular glutathione levels with the resulting ROS increase (Chen et al. 2019).

Pollutants, in particular heavy metals, can also affect muscle strength. Indeed, a recent study followed by Wu and colleagues demonstrates how environmental pollutants can be found in urine of exposed subjects and their levels are inversely related to handgrip strength in children. This suggests that children's muscle strength, normally linked to lack of exercise or reduced energy intake, is strictly connected to environmental pollutants and their local action on muscles. Handgrip strength is a globally used indicator of muscle strength, which if decreased in childhood is related with higher probability to develop during life diseases and conditions such as type 2 diabetes, hypertension, arterial stiffness, and cardiovascular impairment. Mechanisms underlying the association between handgrip strength and heavy metals are not known yet; however, one of the main hypotheses involves ROS generation. In fact, several heavy metals induce ROS production with consequent mitochondria damages and oxidative stress. Moreover, heavy metals seem to have obesogenic effects leading to higher risk to develop obesity. This results in accumulation of intramuscular adipose tissue with a subsequent reduction of muscle strength (Wu et al. 2022). Another environmental factor, acting as an endocrine disrupting chemical, is the tributyltin (TBT), an organotin species used as heat stabilizers, biocide, and as constituent of antifouling paints. It is present in muscle and livers of marine aquatic organism due to the low solubility and lipophilic properties, and it can promote obesogenic and diabetogenic processes, inhibition of myogenesis (downregulating myogenin and myosin heavy chain expression) and multinucleated myotube formation in mice (Chiu et al. 2023).

Environmental pollutants exposure altering muscle physiology can also be linked, in an interplay with genetic factors, to neuromuscular pathologies, such as amyotrophic lateral sclerosis (ALS). This pathology has been recognized to be certainly associated with male sex, age, physical exercise history and tobacco smoking, with the latter two agents being part of the exposome. In particular, cigarette smoke consists of several harmful factors, such as hydrocarbon compounds, pesticides, nitric oxide, and free radicals, that exert detrimental effects on human organism. However, which of these toxic compounds is majorly involved with ALS need to be identified yet. Similarly, many pollutants can be found in the environment, among which plastics, other metals, chemicals and particulates. Although the study of the association between all these substances and nervous system damages has not been examined in depth, some organic pollutants, specifically polybrominated diphenyl esters and polychlorinated biphenyls, seem to be connected to decreased survival in ALS patients. Moreover, in case of genetic predisposition, exposure to environmental pollutants during childhood and adolescence could have an impact on ALS development. Fine particulate PM_2.5_ and PM_<10_ is one of the most dangerous environmental pollutants especially for young people and the adverse effect on human health seems to be related with time of exposure and size of particles. In fact, PM_2.5_ have been proved to increase inflammation and oxidative stress levels, thus leading to gray matter loss and damage to memory (Swash and Eisen 2020).

**Fig. 1:**
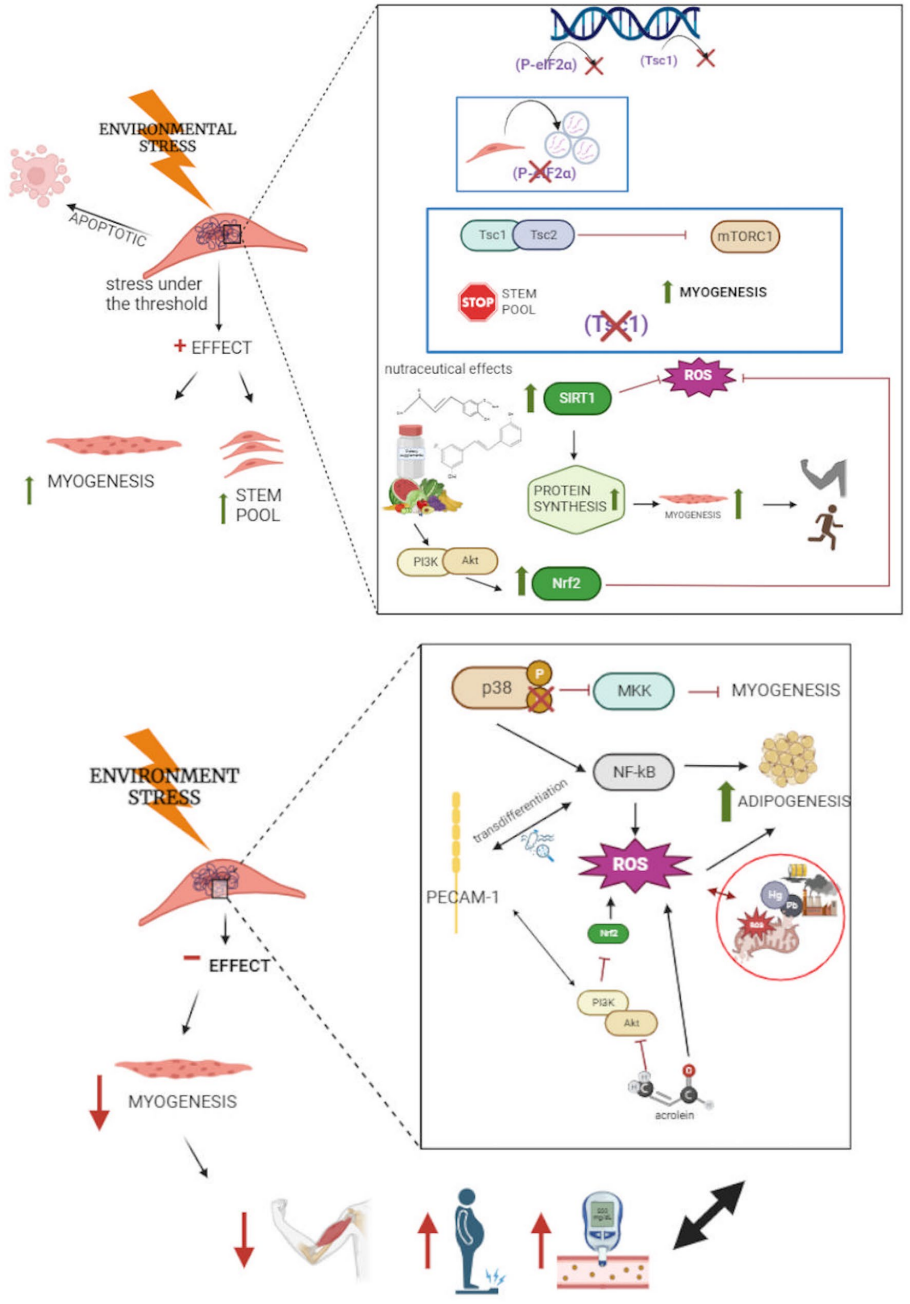
Beneficial (upper panel) vs detrimental (lower panels) effects of environmental stress to the skeletal muscle system, simplified in the binary model characterized by a putative exposure threshold. Image created with BioRender (https://biorender.com)

Taken together, these observations show the impact of the exposome on human health suggesting that further research on the interaction between exposome and neuromuscular pathologies will be of greater interest to address from new perspectives the environmental influence on human organism. In addition, cited observations can explain how the pollutants may have an impact for the establishment of pathological conditions, in a finely regulated interplay with genetic factors (Fig. 1).

## Perspectives

Since millions of commercial, occupational, and environmental chemicals exist, to enable population health studies novel analytical workflows to operationalize untargeted exposomics are needed. Interestingly, a single-step sample-preparation method for use with gas chromatography–high-resolution mass spectrometry has been recently established. Authors stated this method has provided advancements to maximize capture of information on unidentified chemicals from multiple biological matrixes as plasma, lung, thyroid, and stool samples (Hu et al. 2021).

To deal with exposome, one should consider the measurements of both the exogenous factors occurring outside the body and the endogenous doses and biological responses; the advancements toward comprehensive exposure assessment include assessing multiple, co-occurring exposures similar to real-life exposure conditions, understanding the interactions of exposures with endogenous processes and the resulting biological effects, and differentiating exposure windows over the life course (Niedzwiecki et al. 2019). Moving beyond traditional epidemiological studies, Environment-Wide Association Studies (EWAS) allow to analyze a panel of exposures (ecosystems, lifestyle, social factors) in relation to a phenotype of interest, thereby defining a time-varying and dynamic Exposome Risk Score (ERS) through age-related exposures and susceptibilities (Vermeulen et al. 2020). Ever-growing advancements in bioinformatic tools enable an unprecedented acceleration of development in these areas. These advancements may also enable to identify how genetic diversity and ethnic differences influence susceptibility and exposure–susceptibility interactions. Moreover, there is the need to better understand how the effect of exposure to environmental factors are propagated across generations, thereby contributing to multigenerational disease possibly affecting a plethora of tissues (Davis et al. 2022).

For what concerns the biological processes, cells need to sense both the functional situation of the cell and of the surrounding environment; a deeper knowledge of this sensing systems will advance the possibility to evaluate the link exposure factors to the resulting biological effects. Within this framework, mitochondrion has been suggested as the hub of the cross-talk between H_2_O_2_, Ca^2+^, and Zn^2+^, thereby affecting the excitation–contraction-release cycle of skeletal muscle and the onset of peripheral fatigue (Di Filippo et al. 2022). This evidence fits the Mitochondrial Information Processing System (MIPS) perspective: mitochondrial biology can be integrated under the framework of signal transduction of complex adaptive systems; mitochondria are the core of distributed MIPS and are positioned at the interface of the outer and the inner world of the cell, tuning several stress response pathways (Picard and Shirihai 2022).

To counteract the cumulative influences throughout the lifespan that impair stress-responsive cellular pathways, hormetic agents have emerged as any agents capable of improving or at least preserving cellular homeostasis in response to stressors: nutritional factors have been studied on these terms, along with another big pillar of lifestyle, i.e., physical activity (Bevere et al. 2022). It has been argued that an integrated healthy aging strategy that optimizes diet, exercise and other hormetic factors could represent an effective approach for counteracting muscle loss and function (Calabrese and Calabrese 2022).

Diet and exercise can influence and modify gut microbiota. In particular, it seems that physical exercise exerts a beneficial effect on the intestinal microbiota, as well as improving muscle health. Moreover, gut microbiota has been linked to a variety of age-related pathologies: indeed, the literature of the last decades showed the involvement of the so-called "gut–brain axis" in neurodegenerative diseases. Also, it has been seen that gut microbiota is involved in pathophysiological mechanisms that lead to muscle atrophy and sarcopenia. It means that a modulation of microbiota, through lifestyle factors and environmental factors, which allows a healthy microbiota relates to good life’s quality and healthy aging (Strasser et al. 2021).

Moreover, the impact of environmental factors on the aging process must be contemplated. Considering this, it is important, for example, to reduce environmental pollution exposure and improve air quality to decrease the occurrence of musculoskeletal diseases connected with aging process. However, further studies are essential to explain the mechanisms linking pollution and muscle pathologies and to find method to enhance healthy musculoskeletal aging. In fact, exposome can be used as an instrument to better understand and to improve the connection between environmental elements and healthy aging. A first step in this direction is represented by the study of biological markers of aging, among others oxidative stress, mitochondrial function, inflammation, epigenetic modification, telomere length and DNA damages, and of any changes in these markers caused by pollutants (Pandics et al. 2023). In this way, it will be possible to comprehend the role of the exposome in the modulation of the discussed biological processes.

An unprecedented type of exposome has recently emerged, entirely unshaped by evolutionary forces, and which dramatically disrupt muscle mass and function: long-term human spaceflight. Spaceflight-exposome includes primarily microgravity, along with exposure to radiations, harsh workload, disruption of circadian rhythms, isolation and confinement. Capri and colleagues have recently reviewed this topic, thereby reporting that long-term spaceflight or ground-based analog models induce muscle wasting and weakness, incomplete muscle recovery, decrease in costameric-phosphorylated protein FAK-pY397, decrease in desmosomal component, activation of the proteasomal and autophagic-lysosomal pathways, changes in myo-miRNAs (Capri et al. 2023). As authors argued, research fields of aging and human spaceflight could mutually fertilize each other.

This mini-review has shown how cumulative environmental exposures and associated biological responses across the lifespan can affect the skeletal muscle system. With regard to several topics addressed in this manuscript, further research on human models is undoubtedly required to reinforce pivotal concepts and potentially identify novel ones. Findings from this area may guide multiple developments in the field of neuromuscular disease and healthy muscle aging.
